# Nuclear Klf4 accumulation is associated with cetuximab drug-resistance and predicts poor prognosis of nasopharyngeal carcinoma

**DOI:** 10.1186/s12967-018-1561-0

**Published:** 2018-07-04

**Authors:** Xiqing Li, Zunlan Zhao, Shijiang Yi, Lei Ma, Ming Li, Mingyue Liu, Yaping Zhang, Guangzhi Liu

**Affiliations:** 1 Henan Provincial People’s Hospital, 7 Weiwu Road, Zhengzhou, 450003 Henan China; 2 The Health Sciences Biomedical Research Facility II, University of California San Diego, La Jolla, CA 92121 USA; 3 Affiliated Hospital of Guilin Medical University, 1 Lequn Road, Guilin, 541004 Guangxi China; 4 Cancer Hospital of Guangzhou Medical University, 78 Hengzhigang Road, Guangzhou, 510060 Guangdong China; 5 Harvard Medical School, 55 Fruit Street, Boston, MA 02114 USA; 6 University of Illinois at Chicago College of Medicine, 1835 W Polk St, Chicago, IL 60612 USA

**Keywords:** Klf4, Cetuximab, Drug-resistance, Prognosis, Nasopharyngeal carcinoma

## Abstract

**Background:**

The functions of the protein expressed in the nucleus and cytoplasm were different or opposite. The previous study found that oncogene *Klf4* played a role of tumor suppressor in the nasopharyngeal cytoplasm. Cetuximab targeted epidermal growth factor receptor (EGFR) for the treatment of nasopharyngeal carcinoma.

**Methods:**

A cohort of 231 cases of advanced nasopharyngeal carcinoma (7th AJCC III–IVa) samples was assessed by immunohistochemistry (IHC), of which, 63 cases were treated with basic treatment without cetuximab, the basic treatment include chemotherapy and radiotherapy, the regent of the chemotherapy include cisplatin and fluorouracil and 168 cases were treated with cetuximab in addition to the basic treatment. The expression of the KLF4 protein was detected in nucleus and cytoplasm, c-Met protein and nuclear EGFR protein (nEGFR) by IHC, and H-Ras and PI3K mutations by an arms-PCR method in vivo. KLF4 was found to specifically express in the cytoplasm by deleting the NES, while *H*-*Ras* and *PI3K* genes were mutated in the nasopharyngeal carcinoma 5–8F and HONE1cell line. The cetuximab resistance in differentially mutated 5–8F and HONE1 cells was analyzed.

**Results:**

The expression of *Klf4* in the nucleus was associated with prognosis in 168 patients with cetuximab-treated nasopharyngeal carcinoma, which was found by retrospective analysis. The KLF4 expression in the nucleus was not significantly correlated with the prognosis in 63 nasopharyngeal carcinoma patients treated with basic treatment (P = 0.261). The expression of *Klf4* in the nucleus was correlated with mutations of H-Ras and PI3K in 168 cases of nasopharyngeal carcinoma with cetuximab treatment. In vitro experiments showed that *Klf4* was specifically expressed in the nucleus of 5–8F and HONE1 cells as assessed by deleting nuclear export signal, which led to cetuximab resistance. H-Ras and PI3K mutations in 5–8F and HONE1 cells also led to the expression of *Klf4* in the nucleus and resistance to cetuximab. In HONE1 cells, Klf4 was specifically localized in the cytoplasm by deleting the NES, and the H-Ras and PI3K mutations did not result in an increased expression of Klf4 in the nucleus and cetuximab resistance.

**Conclusion:**

The prognosis of nasopharyngeal carcinoma was not significantly improved by cetuximab treatment when the *Klf4* was highly expressed in the nucleus of nasopharyngeal carcinoma tissues. The expression of *Klf4* in the nucleus can be used as a biomarker for predicting the effects of cetuximab treatment in nasopharyngeal carcinoma, which might be attributed to the H-RAS and PI3K mutations, leading to cetuximab resistance.

## Background

Presently, cetuximab is the only molecular targeted drug approved for the head and neck cancer treatment in clinical practice. It is a human–mouse chimeric antibody that blocks the binding of EGFR [1]. The development and progression in 90% nasopharyngeal carcinomas are caused by EGFR. Theoretically, the use of cetuximab to block EGFR has inhibitory effects in most of the nasopharyngeal carcinomas. Moreover, a large proportion of head and neck tumors are resistant to cetuximab. Only 13% of the head and neck tumors benefitted from the use of cetuximab [2]. Despite multiple assessments of the resistance-related factors to cetuximab, no consensus marker has been established for the prediction of therapeutic effects of cetuximab in the treatment of head and neck cancer. A large number of molecular markers associated with cetuximab resistance were identified with respect to the nuclear expression of EGFR, H-Ras mutation, PI3K mutation, and c-Met protein overexpression [3–5].

Nasopharyngeal carcinoma differed from the common head and neck tumors. It was primarily caused by the EB virus but not caused by HPV in the other head and neck cancers [6]. The nasopharyngeal carcinoma tissue has more keratinization and intercellular bridges than the other head and neck cancers. Thus, specific analysis and study of cetuximab resistance in nasopharyngeal carcinoma is essential. However, the efficiency of cetuximab in the treatment of nasopharyngeal cancer was low [7]. As a common high-incidence tumor in southern China, cetuximab has been shown to prolong the overall survival for only 3 months in some patients with nasopharyngeal carcinoma. Thus, identifying the efficiency of cetuximab treatment in patients was essential [8].

*Klf4* was commonly expressed in nasopharyngeal carcinoma. Previously, KLF4 was found to be expressed in the cytoplasm of nasopharyngeal carcinoma, and it played a role as tumor suppressor gene [9]. Studies have shown that KLF4 was associated with H-Ras and PI3K, which are downstream to the EGFR pathway, mutations in the Ras pathway regulate the KLF4 expression [10], the PI3K mutation was accompanied by altered KLF4 expression [11]. H-Ras and PI3K were common mutations associated with cetuximab resistance, and contrary to our previous research, KLF4 played the role of an tumor suppressor in these processes. The different functions of KLF4 were associated with its subcellular localization in cells and structure. The structural conformation of *Klf4* had three typical C-terminus C2H2 zinc fingers (ZFs) as DNA binding regions and N-terminus activation regions [12], in the study of KLF4, nuclear localization sequence (NLS) has been shown to play a role in subcellular localization and is specifically expressed in the cytoplasm or nucleus by modifying the nuclear exporting sequence (NES) [12], and then analyze the relationship between different subcellular localizations and cetuximab resistance.

## Methods

### Clinical case data and tumor specimens

A total of 231 cases of nasopharyngeal carcinoma tissue specimens were selected by biopsy. All the patients were middle and advanced nasopharyngeal carcinoma (7th AJCC III–IVa); 63 were not treated with cetuximab but only treated with conventional radiotherapy and chemotherapy, and 168 were treated with cetuximab according to a standardized treatment. No patients with nasopharyngeal carcinoma underwent chemotherapy and radiotherapy before sample biopsy. In these specimens, 105 cases were collected at the Guangzhou Cancer Hospital from 2007 to 2012, 73 from Affiliated Hospital of Guilin Medical College from 2007 to 2013, and 53 from People’s Hospital of Zhengzhou University from 2009 to 2013. This study was approved by the Ethics Committee of Zhengzhou University People’s Hospital.

### Immunohistochemistry (IHC) method

After deparaffinization and rehydration, the paraffin-embedded sections were subjected to antigen retrieval for 2 min. Then, the slides were incubated overnight at 4 °C with the following primary antibodies: (dilution 1:200, abcam, MA). Serial tumor sections and IHC staining evaluated the expression of proteins. KLF4 immunoreactivity was examined using a mouse polyclonal antibody. PBS was used as a negative control by omitting the primary antibody. The avidin–biotin technique was applied using DAB for visualization and hematoxylin for counterstaining. Histological and IHC evaluation were independently performed by two pathologists, who were blinded to the clinicopathological outcomes of the patients. Briefly, each slide was examined under a light microscope, and an initial score was assigned, which represented the estimated proportion of positive tumor cells (0: ≤ 5%; 1: 5–25%; 2: 25–75%; 3: ≥ 75%). The scores 0, 1 were defined as low and 2, 3 as high. The slides with indeterminate evaluation were re-evaluated, and a consensus was reached, as described previously [9].

### Mutation analysis of PI3K and H-Ras

Mutations in *PI3K* and *H*-*RAS* genes were detected in the clinical nasopharyngeal carcinoma specimens. 50 mg tissue specimens were selected from pathologically confirmed nasopharyngeal carcinoma tissues, and QIAamp DNA extraction kit (Shanghai, China) was used. Mutations in the first and second exons of *H*-*RAS* were detected. The forward primer for exon detection was hras1f-AGACCCTGTAGGAGGACC, and the reverse primer for the first exon was hras1r-GAGGAAGCAGGAGACAGG, while the forward primer for the second exon was HRAS-E1-R-CTCGCCCGCAGCAGCTGCTG, and the reverse primer was HRAS-E2-R-GGGCCAGCCTCACGGGGTTC. The PCR products were analyzed by gel electrophoresis, and the products extracted and amplified for sequencing [13]. For the *PI3K* gene, the mutations in the exons 9 and 20 were detected, and the primers were designed as described previously [14]. The sequencing primers for HNSCC-associated *PIK3K* hotspot mutations were synthesized (Sigma-Aldrich, St. Louis, MO, USA) and used for Sanger sequencing. The primer sequences for *E542* site mutation were: 5′-cacgagatcctctctctaaaatcactgagcaggag-3′ (forward) and 5′-ctcctgctcagtgattttagagagaggatctcgtg-3′ (reverse). Sanger sequencing was performed at the Genomics and Proteomics Core Laboratories at the University of Pittsburgh, USA [14].

### Nuclear protein localization and expression

We demonstrate using EGFP fusion constructs that KLF4 nucleo-cytoplasmic transport is not regulated by the 5′ basic region but activated by a novel NLS and a nuclear export signal (NES), we demonstrate KLF4 nuclear export to be Crm1-dependent. Our previous prediction by bioinformatics software found that the NLS of KLF4 was localized at 404–417 aa (PKPKRGRRSWPRKR) and NES was located at 90–100 aa (FNDLLDLDFIL). The preliminary validation showed that the NES sequence could induce the localization and expression of KLF4 in the nucleus. The NLS (PredictNLS, https://rostlab.org/owiki/index.php/PredictNLS/) and the NES sequences (NetNES 1.1 Server, http://www.Cbs.dtu.dk/services/NetNES/) of KLF4 were predicted by bioinformatics and confirmed. The NLS and NES oligonucleotide sequences were synthesized. The EGFP fusion expression vector pEGFP-C1-Klf4 was constructed. Site-directed mutagenesis was employed for deleting the NLS and NES sequences, and pEGFP-C1-Klf4-del NLS and pEGFP-C1-Klf4-del NES vectors were constructed, respectively. Fluorescence microscopy displayed the localization of KLF4 after transfection into nasopharyngeal carcinoma cells to verify the plasma and nuclear localization.

### Construction of HONE1 cells with H-Ras and PI3K mutations

The *H*-*Ras* and *PI3K* genes were mutated by using vectors. In order to detect whether the mutations of H-Ras and PI3K in nasopharyngeal carcinoma HONE1 cells would affect the expression of KLF4 and the therapeutic sensitivity of cetuximab to nasopharyngeal carcinoma cells, we firstly constructed the vectors with H-Ras mutation and the corresponding control. pBabe-HRAS G12D or pBabe-HRAS G12V vectors were used to construct the H-Ras mutant constructs. Moreover, the vectors of wild-type H-Ras and H-Ras G12D were constructed as controls. These vectors were purchased from Addgene. The mutations in the gene fragments were constructed using the QuikChange site-directed mutagenesis kit (Stratagene, MA). All constructs were authenticated by DNA sequencing [15]. The PI3K mutants in exons 9 and 20 were constructed using the N-FLAG-tag PIK3CA expression vector and the point mutation kit. The constructed plasmids were sequenced and purified for transfection into the cells [13].

### Immunofluorescence

Immunofluorescence was used to observe the expression location of KLF4 in the cells. The HONE1cells were cultured in a slide-added culture dish to allow the cell growth on glass slides. Subsequently, the cells were fixed with 4% paraformaldehyde, followed by hydrated with PBS and incubation with KLF4 antibody for 45 min. Then, secondary Alexa Fluor 488-conjugated anti-rabbit antibody was reacted for 45 min at room temperature. The nuclei were stained with DAPI. Finally, the slides were observed under a confocal microscope to detect the location of KLF4 based on the fluorescence intensity (Olympus FV1000, Japan) [16].

### Western blot

Cytoplasmic and nuclear proteins of HONE1 cells were extracted by Nuclear and Cytoplasmic Protein Extraction Kit (Beyotime), protein lysates were separated by sodium dodecyl sulfate–polyacrylamide gel electrophoresis (SDS-PAGE) and transferred to a polyvinylidene difluoride (PVDF) membrane. The blots were probed with the primary antibodies against Klf4, followed by HRP (horseradish peroxidase)-labeled secondary antibodies. The hybridization signal was detected using enhanced chemiluminescence (ECL). GAPDH was used as a loading control [9].

### Apoptosis was detected by flow cytometry

The adherent 5–8F and HONE1 cells were trypsinized, and then resuspended in binding buffer. Then, Caspase 3/7 was added to the mixture and incubated in the dark for 25 min at room temperature. PI was added to the cells 5 min before flow cytometry. The results of flow cytometry detection were analyzed by Flow Jo (BD bio, CA).

### Statistical analysis

Statistical analysis was performed using the SPSS 13.0 software package. Associations between the clinicopathological features and Klf4 and nEGFR expression by IHC were analyzed using the Chi squared test. Multivariate survival analyses were performed using the Cox regression model. The overall survival (OS) was measured from the onset of treatment to the date of death or the survival status at the last date of follow-up. The OS probabilities were estimated by the Kaplan–Meier method and the significance of differences was assessed by the log-rank test. The correlations between Klf4 and nEGFR expression and/or H-Ras and PI3K mutation with clinicopathological factors were analyzed using Fisher’s exact probability test or the Chi squared test. *P* value < 0.05 was considered as statistically significant.

## Results

### Expression of KLF4 in the nucleus induced a poor prognosis in patients treated with cetuximab

**Fig. 1:**
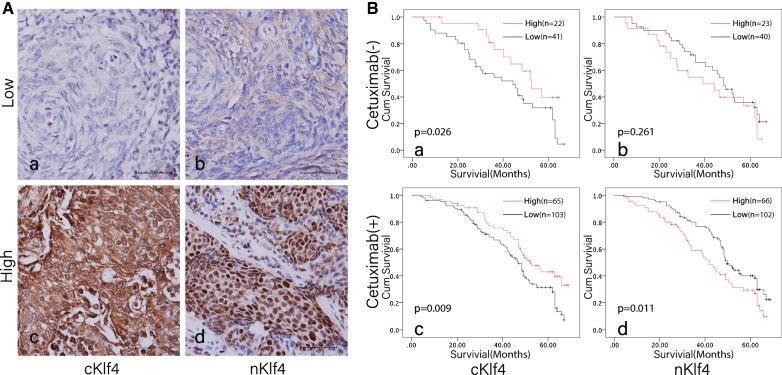
KLF4 expressed in the nucleus had a relatively poor prognosis in patients treated with cetuximab. **A** Representative image of the IHC detection of KLF4 protein expression in nasopharyngeal carcinoma. **A**-**a** Low expression of KLF4 in the cytoplasm of nasopharyngeal carcinoma. **A**-**c** KLF4 was highly expressed in the cytoplasm. **A**-**b** Low expression of KLF4 in the nucleus of nasopharyngeal carcinoma cells. **A**-**d** KLF4 was highly expressed in the nucleus. **B** KLF4 in the survival analysis of nasopharyngeal carcinoma. **B**-**a** In nasopharyngeal carcinoma patients treated with cetuximab, the prognosis of patients with highly expressed cytoplasmic KLF4 was significantly better than that in those with low expression. **B**-**d** The nuclear expression of KLF4 had no significant effect on the prognosis in the patients without cetuximab treatment. **B**-**c** In the nasopharyngeal carcinoma patients treated with cetuximab, the expression of KLF4 in the cytoplasm had a better effect on the prognosis. **B**-**d** The expression of KLF4 in the nucleus had a relatively poor effect on the prognosis in the patients combine with cetuximab treatment

Tumor specimen from 168 NPC patients consisting of 94 males and 74 female were included in this study. The median age was 54 years (range 34–79 years), clinicopathological features as shown in Table 1. The expression of KLF4 in the nucleus and/or cytoplasm of nasopharyngeal carcinoma tissues was detected by IHC in Fig. 1A is the representative image). The two groups patients were as follows: one treated with cetuximab in addition to basic treatment (168 cases) and the other is only basic treatment without cetuximab treatment (63 cases). The relationship between KLF4 expression and the general clinical parameters in 168 cases of nasopharyngeal carcinoma with cetuximab treatment in addition to basic treatment was shown in Table 1. The KLF4 was highly expressed in the cytoplasm of 22 cases of 63 cases without cetuximab treatment. However, no significant correlation was established between the expression of KLF4 and age, sex, and clinical stage in the 63 cases. Patients with basic treatment only showed a high expression of the KLF4 protein in the cytoplasm with a better prognosis than that of the low expression (Fig. 1B-a, P = 0.026), correspondingly, 23 cases showed a high expression of KLF4 in the nucleus but without a significant correlation with prognosis (Fig. 1B-a, P = 0.261). Moreover, in the 168 cases treated with cetuximab in addition to basic treatment, 65 showed a high expression of KLF4 in the cytoplasm. The prognosis of patients with high-expression of KLF4 in the cytoplasm was significantly better than that in the low-expression group (P = 0.009). Furthermore, of the 168 cases of nasopharyngeal carcinoma patients, 56 showed a high expression of KLF4 in the nucleus that can cause poor prognosis of nasopharyngeal carcinoma (P = 0.011).

**Table 1:** The clinicopathologic characteristics of nKlf4 and cKlf4 expression in NPC patients

Variables	Cases	nKlf4 (n, %)			cKlf4 (n, %)		
		Low	High	*P*	Low	High	*P*
Gender							
Male	94	63 (67.0)	31 (33.0)	0.059	53 (56.4)	41 (43.6)	0.139
Female	74	39 (52.7)	35 (47.3)		50 (67.6)	24 (32.4)	
Age (years)							
< 54	87	53 (60.9)	34 (39.1)	0.955	55 (63.2)	32 (36.8)	0.599
≥ 54	81	49 (60.5)	32 (39.5)		48 (59.3)	33 (40.7)	
T classification							
T3	24	14 (58.3)	10 (41.7)	0.796	14 (58.3)	10 (41.7)	0.746
T4	144	88 (61.1)	56 (38.9)		89 (61.8)	55 (38.2)	
N classification							
N0	87	56 (64.4)	31 (35.6)	0.315	51 (58.6)	36 (41.4)	0.458
N1–3	81	46 (56.8)	35 (43.2)		52 (64.2)	29 (35.8)	
M classification							
M0	122	76 (62.3)	46 (37.7)	0.494	69 (56.6)	53 (43.4)	0.039
M1	46	26 (56.5)	20 (43.5)		34 (73.9)	12 (26.1)	
Clinical stage							
III	49	36 (73.5)	13 (26.5)	0.030	24 (49.0)	25 (51.0)	0.035
IV	119	66 (55.5)	53 (44.5)		79 (66.4)	40 (33.6)

### KLF4 expression in the nucleus was correlated to H-Ras and PI3K mutations

**Fig. 2:**
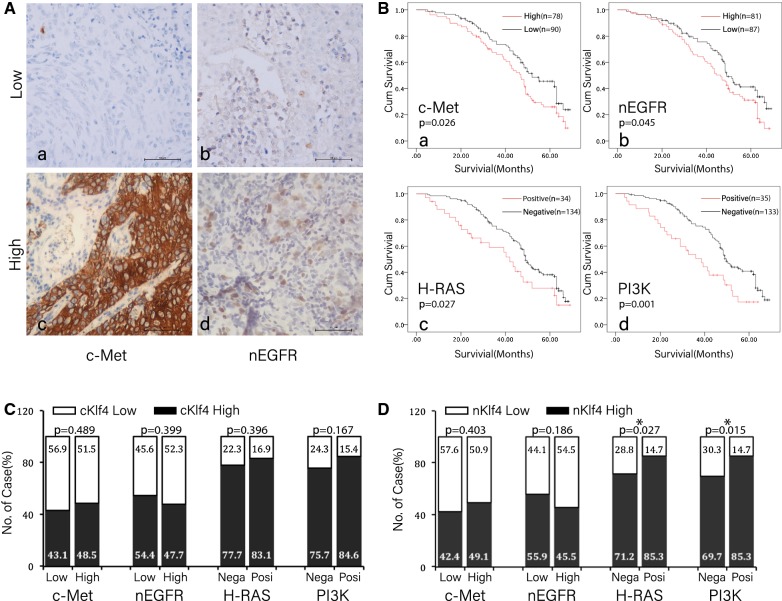
The expression of KLF4 in the nucleus was significantly correlated with H-Ras and PI3K mutations. IHC was used to detect the expression of c-Met protein. EGFR was expressed in the nucleus (nEGFR), and H-Ras mutation and PI3K mutation exerted a relatively poor effect on the prognosis in patients treated with cetuximab. **A**-**a** Low expression of c-Met in nasopharyngeal carcinoma. **A**-**b** Representative image of the high expression of c-Met in nasopharyngeal carcinoma tissues. **A**-**c** Representative imaged the low expression of nEGFR in the nucleus. **A**-**d** Representative image of high-expression of nEGFR in nasopharyngeal carcinoma. **B**-**a** Survival analysis showed that the expression of c-Met had a relatively poor effect on prognosis in cetuximab-treated nasopharyngeal carcinoma patients. **B**-**b** nEGFR expression had a relatively poor effect on prognosis; nasopharyngeal carcinoma patients with mutated H-Ras (**B**-**c**) and PI3K (**B**-**d**) mutations had a relatively poor prognosis after cetuximab treatment. **C** The cytoplasmic cKlf4 expression was not significantly correlated with the expression of c-Met, nEGFR, H-Ras, and PI3K mutations in nasopharyngeal carcinoma tissues, Nega short for negative and Posi short for positive. **D** The expression of KLF4 in the nucleus was significantly correlated with the expression of H-Ras and PI3K mutations

The c-Met and nEGFR protein was detected by IHC in 168 cases of nasopharyngeal carcinoma patients treated with cetuximab and clinicopathological features as shown in Table 2. H-Ras and PI3K mutations were detected by arms-PCR and clinicopathological features as shown in Table 3. The c-Met protein was predominantly expressed on the cell membrane (Fig. 2A-c), while the nEGFR protein was expressed on the cell membrane as well as in the nucleus. This study mainly observed the EGFR protein that was expressed in the nucleus (Fig. 2A-d). The relationship between these indicators and the general clinical parameters was shown in Table 2. 78/168 cases of nasopharyngeal carcinoma highly expressed the c-Met protein. The prognosis of nasopharyngeal carcinoma in the group of high c-Met protein expression was significantly poorer than that of the low expression group (Fig. 2B-a, P = 0.045). The prognosis of nasopharyngeal carcinoma in the group of high nEGFR expression was significantly lower than that of the low expression group (Fig. 2B-b, P = 0.001). H-Ras and PI3K mutations can lead to poor prognosis in patients with nasopharyngeal carcinoma (P = 0.045, P = 0.001). The analysis of the correlation between the KLF4 expression in nasopharyngeal carcinoma and the expression of c-Met and nEGFR, or the correlation between KLF4 expression and H-Ras and PI3K mutations did not identify an association between the cytoplasmic KLF4 expression and the expressions of c-Met and nEGFR and Ras and PI3K mutations. The KLF4 expression in the nucleus was significantly correlated with H-Ras and PI3K mutations. In the nasopharyngeal carcinoma with H-Ras and PI3K mutations, the level of nuclear KLF4 was increased, and the difference was statistically significant (P < 0.05). No significant correlation was observed between the cytoplasmic KLF4 expression and the common indicators of cetuximab resistance. In a multivariate analysis incorporating all clinicopathologic variables and covariates as shown in Table 4.

**Table 2:** The clinicopathologic characteristics of nEGFR and cMet expression in NPC patients

Variables	Cases	nEGFR (n, %)			cMET (n, %)		
		Low	High	*P*	Low	High	*P*
Gender							
Male	94	46 (48.9)	48 (51.1)	0.405	40 (42.6)	54 (57.4)	0.868
Female	74	41 (55.4)	33 (44.6)		32 (43.8)	41 (56.2)	
Age (years)							
< 54	87	41 (47.1)	46 (52.9)	0.210	35 (40.7)	51 (59.3)	0.516
≥ 54	81	45 (56.8)	35 (43.2)		37 (45.7)	44 (54.3)	
T classification							
T3	24	13 (54.2)	11 (45.8)	0.801	14 (58.3)	10 (41.7)	0.104
T4	144	74 (51.4)	70 (48.6)		58 (40.6)	85 (59.4)	
N classification							
N0	87	41 (47.1)	46 (52.9)	0.210	36 (41.9)	50 (58.1)	0.736
N1–3	81	46 (56.8)	35 (43.2)		36 (44.4)	45 (55.6)	
M classification							
M0	122	63 (51.6)	59 (48.4)	0.951	54 (44.3)	68 (55.7)	0.622
M1	46	24 (52.2)	22 (47.8)		18 (40.0)	27 (60.0)	
Clinical stage							
III	49	26 (53.1)	23 (46.9)	0.832	20 (40.8)	29 (59.2)	0.699
IV	119	61 (51.3)	58 (48.7)		52 (44.1)	66 (55.9)

**Table 3:** The clinicopathologic characteristics of nKlf4 and cKlf4 mutation in NPC patients

Variables	Cases	PI3K (n, %)			H-Ras (n, %)		
		Negative	Positive	*P*	Negative	Positive	*P*
Gender							
Male	94	79 (84.0)	15 (16.0)	0.120	78 (83.0)	16 (17.0)	0.242
Female	74	55 (74.3)	19 (25.7)		56 (75.7)	18 (24.3)	
Age (years)							
< 54	87	70 (80.5)	17 (19.5)	0.816	71 (81.6)	16 (18.4)	0.537
≥ 54	81	64 (79.0)	17 (21.0)		63 (77.8)	18 (22.2)	
T classification							
T3	24	21 (87.5)	3 (12.5)	0.308	18 (75.0)	6 (25.0)	0.531
T4	144	113 (78.5)	31 (21.5)		116 (80.6)	28 (19.4)	
N classification							
N0	87	75 (86.2)	12 (13.8)	0.031	73 (83.9)	14 (16.1)	0.166
N1–3	81	59 (72.8)	22 (27.2)		61 (75.3)	20 (24.7)	
M classification							
M0	122	98 (80.3)	24 (19.7)	0.766	97 (79.5)	25 (20.5)	0.894
M1	46	36 (78.3)	10 (21.7)		37 (80.4)	9 (19.6)	
Clinical stage							
III	49	41 (83.7)	8 (16.3)	0.418	38 (77.6)	11 (22.4)	0.647
IV	119	93 (78.2)	26 (21.8)		96 (80.7)	23 (19.3)

**Table 4:** Univariate and multivariate analysis for clinicopathologic variables

Parameters	HR	Univariate analysis		HR	Multivariate analysis	
		95% CI	*P* ^a^		95% CI	*P* ^a^
Gender						
Male vs. female	0.889	0.763–2.111	0.346			
Age (years)						
< 52 vs. ≥ 52	0.385	0.753–2.217	0.535			
T classification						
T3 vs. T4	1.635	1.413–2.299	0.201			
N classification						
N0 vs. N1–3	2.453	0.916–2.054	0.117			
M classification						
M0 vs. M1	1.745	0.761–1.777	0.187			
C classification						
III vs. IV	0.337	1.244–2.170	0.562			
nKlf4						
Low and high	1.616	1.106–2.361	0.013	0.608	0.406–0.909	0.015
cKlf4						
Low and high	0.591	0.395–0.884	0.056	0.596	0.398–0.892	0.027
nEGFR						
Low and high	1.461	1.002–1.920	0.049	1.534	1.050–2.239	0.027
cMET						
Low and high	1.254	0.744–1.734	0.043	1.324	0.773–1.875	0.032
PI3K						
Positive and negative	3.029	2.430–3.628	0.034	1.236	0.544–1.928	0.000
H-Ras						
Positive and negative	1.664	1.050–2.278	0.030	1.298	0.768–1.828	0.000

*HR* hazard rate, *CI* confidence interval

^a^Cox regression model

### Increased expression of KLF4 in the nucleus led to cetuximab resistance

**Fig. 3:**
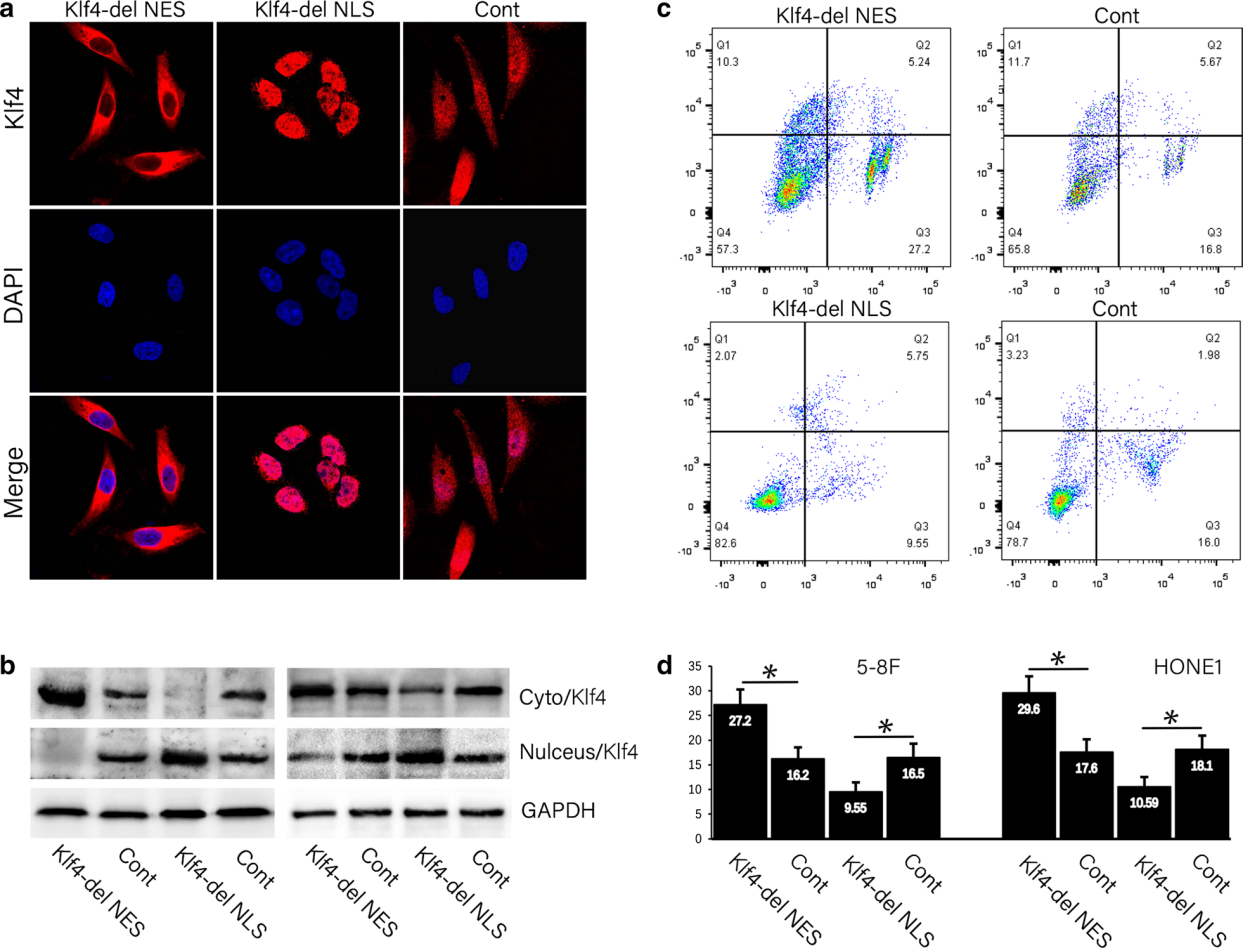
Increased KLF4 expression in the nucleus led to cetuximab resistance. **a** Immunofluorescence confirmed that KLF4 was highly expressed in the cytoplasm of in Klf4-del NES cells, and KLF4 was overexpressed in the nucleus when KLF4 was locally expressed in the nucleus of Klf4-del NLS cells. **b** The nuclear and cytoplasmic proteins in different HONE1 cells were extracted separately, and the expression of KLF4 protein in the nucleus and cytoplasm was verified by Western blot. **c** The Klf4-del NES 5–8F and HONE1 cells were treated with cetuximab, and the apoptosis was affected significantly, meanwhile the apoptosis of cKLF4-HONE1 cells was significantly reduced after treatment with cetuximab (**d**)

Immunofluorescence confirmed that the KLF4 was overexpressed in the cytoplasm of HONE1 cells (Fig. 3a). Alternatively, the Klf4-del NLS cell line with KLF4 specifically expressed in the nucleus was generated by deleting the cytoplasmic localization sequence using pEGFR-C1-Klf4-delNLS. Immunofluorescence showed that the expression of KLF4 was markedly enhanced in the nucleus (Fig. 3a). In order to confirm that the expression of KLF4 protein in the cytoplasm or nucleus was increased, the nuclear and cytoplasmic proteins were extracted, respectively, for detection by Western Blot. The 5–8F (left) and HONE1 (right) showed that the cytoplasmic KLF4 protein was significantly increased in the Klf4-del NES cells as compared to the control group (Fig. 3b) and quantified with a statistical representation of the results in Table 5. Similarly, KLF4 expression was significantly increased in HONE1 nuclei in Klf4-del NLS cells. It is showed the extraction of nuclear and cytoplasmic proteins of 5–8F and HONE1 cells in nasopharyngeal carcinoma. Western blot verified the expression of KLF4 protein in nucleus and cytoplasm. The cells were treated with 2 μg/mL cetuximab after high expression of KLF4 in the cytoplasm and nucleus. Flow cytometry was used for the detection of apoptosis (Fig. 3c) and revealed that cetuximab was induced apoptosis in HONE1 cells (Q2 indicated apoptotic cells) as compared to the control group. Compared to the HONE1 cells with transfection of control plasmid, the cetuximab-induced apoptosis in the cells with high expression of KLF4 in the cytoplasm was not increased significantly. Correspondingly, after high expression of KLF4 in the nucleus, the apoptosis was decreased significantly in cetuximab-treated cells as compared to 5–8F and HONE1 cells with transfection of control plasmid, suggesting resistance to cetuximab (Fig. 3d).

**Table 5:** Quantified the value of bands of Western blot (Int)

	Klf4-del NES	Cont	Klf4-del NLS	Cont	*P* value
Cyto/Klf4	100,584.55 ± 8594.65	67,233.35 ± 8919.09	5932.65 ± 4147.62	58,734.76 ± 947.97	< 0.001
Nulceus/Klf4	3048.7 ± 1094.65	31,620.08 ± 7840.48	28,007.37 ± 8226.13	10,582.34 ± 9782.6	< 0.001
GAPDH	55,672.06 ± 4284.88	54,125.94 ± 5084.87	53,428.41 ± 6540.34	61,599.02 ± 17,604.66	0.763
Cyto/Klf4	96,878.2 ± 7884.04	79,753.3 ± 5296.64	56,818.47 ± 8976.7	72,769.22 ± 7592.35	< 0.001
Nulceus/Klf4	14,781.7 ± 4647.97	45,721.43 ± 3199.21	71,261.5 ± 3312.42	53,996.92 ± 9576.89	< 0.001
GAPDH	41,617.74 ± 3302.21	49,779.71 ± 9021.16	49,104.88 ± 1985.12	52,330.03 ± 786.79	0.873

### Mutations of *H*-*Ras* and *PI3K* genes led to increased KLF4 expression in the nucleus and cetuximab resistance

**Fig. 4:**
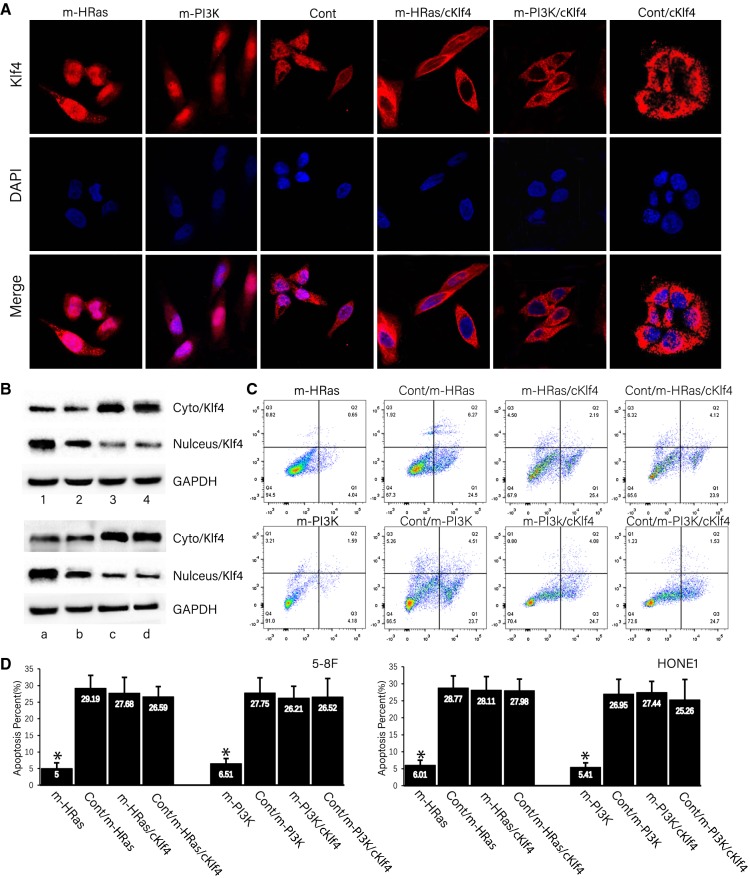
KLF4 expression in the nucleus is related to H-Ras and PI3K mutation. **A** Immunofluorescence demonstrated that KLF4 expression was increased in mHRas HONE1 cell and m-PI3K HONE1 cells in nasopharyngeal carcinoma cell line HONE1, respectively. The expression of KLF4 was not increased in the cytoplasm in m-HRas/cKlf4 HONE1 cells and mPI3K/cKlf4 HONE1 cells. **B** WB demonstrated that the expression of KLF4 was increased in the nucleus of m-Hras HONE1 cells (2) and m-PI3K HONE1 cells (b) and no change was observed in KLF4 in the cytoplasm and nucleus in mHRas/cKlf4 HONE1 cells (3) and m-PI3K HONE1 cells (c), Cyto/Klf4 is an abbreviation for protein extraction from the cytoplasm, and Nulcelus/Klf4 is an abbreviation for protein extraction from the nucleus. **C** m-HRas HONE1 cells and m-PI3K HONE1 cells were resistant to cetuximab, and the experimental group m-HRas/cKlf4 HONE1 cells and mPI3K/cKlf4 HONE1 cells did not cause the resistance to cetuximab as compared to the control group that had high expression of KLF4 in the cytoplasm. **D** m-HRas 5–8F or HONE1 cells, m-PI3K 5–8F or HONE1 cells, m-HRas/cKlf4 5–8F or HONE1 cells, and m-PI3K 5–8F or HONE1 cells were treated with cetuximab, and cetuximab did not significantly caused apoptosis in m-HRas 5–8F or HONE1 cells and m-PI3K-5–8F or HONE1 cells

HONE1 cells with H-Ras mutation (abbreviated as m-HRas) and HONE1 cells with PI3K mutation (abbreviated as m-PI3K) were established, and immunofluorescence microscopy was performed to investigate the expression location of KLF4. The KLF4 fluorescence intensity was found to be increased in the nucleus of H-Ras mutation cells and PI3K mutation cells. Moreover, the mutations in *H*-*Ras* and *PI3K* genes were effectuated in Klf4-del NES cells with specifically expressed KLF4 in the cytoplasm. And then, the m-HRas/cKlf4 cells with KLF4 expression in the cytoplasm and H-Ras mutation, and m-PI3K cells with KLF4 expression in the cytoplasm were also constructed (m-PI3K/cKlf4 cells). Immunofluorescence showed that the expression of KLF4 was increased in the cytoplasm in m-HRas/cKlf4 and m-PI3K/cKlf4 cells, meanwhile, the KLF4 expression in the nucleus was not affected by H-Ras and PI3K mutations with respect to the cytoplasm in HRas/cKlf4 and m-PI3K/cKlf4 cells (Fig. 4A). Western blot verified that KLF4 protein expression in the nucleus of m-HRas Hone1 cells (#1) was increased as compared to that of the control group (#2). The KLF4 protein expression in the nucleus of mHRas/cKlf4 HONE1 cells (#3) was not altered significantly as compared to the control group (#4) (Fig. 4B, up), similarly, KLF4 protein expression in the nucleus of m-PI3K Hone1 cells (#a) was increased as compared to that of the control group (#b). The KLF4 protein expression in the nucleus of mPI3K/cKlf4 HONE1 cells (#c) was not altered significantly as compared to the control group (#d) (Fig. 4B, down) and and quantified with a statistical representation of the results in Table 6. Eight different genetically modified HONE1 cells—mHRas, Cont/m-HRas, m-HRas/cKlf, Cont/m-HRas/cKlf4, mPI3K, Cont/m-PI3K, m-PI3K/cKlf, Cont/m-PI3K/cKlf4—were treated with cetuximab. The m-HRas and m-PI3K in HONE1 cells were found to be resistant to cetuximab after the mutation of *H*-*Ras* and *PI3K* genes, with a significant reduction of cetuximab-induced early apoptosis (Q2) (Fig. 4C). In the specific expression of KLF4 protein in the cytoplasm HONE1 cells, after m-HRas and m-PI3K mutation were not significantly resistant to cetuximab as compared to the control group. The changes in the ratio of apoptotic cells treated with cetuximab were analyzed in 5–8F and HONE1 cells with four different genetic alterations, respectively. The early apoptosis of m-HRas and m-PI3K in 5–8F and HONE1 cells were significantly decreased as compared to Cont/m-Hras and Cont/m-PI3K cells. Correspondingly, the early apoptosis of m-HRas/cKlf4 and m-PI3K/cKlf4 cells were no significantly increased as compared to control cells (Fig. 4D).

**Table 6:** Quantified the value of bands of Western blot (Int)

	1	2	3	4	*P* value
Cyto/Klf4	48,159.69 ± 8123.32	33,548.97 ± 7378.29	91,026.73 ± 7402.96	95,467.39 ± 336.03	< 0.001
Nulceus/Klf4	86,629.46 ± 5175.7	53,506.9 ± 7446.03	24,095.91 ± 6497.69	21,932.73 ± 7842.6	< 0.001
GAPDH	58,029.14 ± 2057.48	53,464.83 ± 7513.77	60,053.84 ± 6565.42	63,457.21 ± 433.29	0.871

	a	b	c	d	*P* value
Cyto/Klf4	5480.6 ± 4561.18	43,422.75 ± 7581.51	84,011.77 ± 6633.16	87,969.27 ± 4280.41	< 0.001
Nulceus/Klf4	81,021.94 ± 9163.29	63,380.68 ± 7649.24	33,969.69 ± 6700.9	32,095.06 ± 7686.94	< 0.001
GAPDH	51,054.26 ± 4366.05	53,338.61 ± 7716.98	53,927.62 ± 6768.64	49,833.77 ± 9560.99	0.658

## Discussion

Drug resistance is common in the treatment of molecular-targeted drugs; for example, T790M mutation of EGFR in lung adenocarcinoma was the major cause of gefitinib resistance and other TKI drugs’ resistance [17], while K-Ras mutation was the main cause of cetuximab resistance in colorectal cancer [18]. The molecular targeted drug therapy theoretically stated that the potentially effective patients could be screened out by biological markers, which could improve the rate of efficiency in patients for accurate treatment, thereby providing additional treatment opportunities for cancer patients.

A number of indicators have been reported in the head and neck tumors to predict the resistance of cetuximab, such as EGFR expression [19], EGFR gene polymorphism [4], EGFR VIII expression [20], EGFR expression in the nucleus [3], methylation of EGFR [21], ErbB pathway and MET activation [22], and Axl overexpression [23] and changes in the downstream pathway of EGFR, such as HRAS mutation, deletion of *PTEN* gene [24], and abnormalities in the PI3K pathway [25]. Because of the differences between nasopharyngeal carcinoma and head and neck tumors, and the unique biological characteristics of nasopharyngeal carcinoma, the cetuximab resistance might be predicted in the head and neck tumors; however, whether there was a correlation between these indicators and the drug resistance of cetuximab therapy in nasopharyngeal carcinoma has not been analyzed [26].

In previous studies, we verified the several common indicators related to cetuximab resistance in nasopharyngeal carcinoma. c-MET, nEGFR, H-Ras, and PI3K mutations [3] were also related to cetuximab resistance in nasopharyngeal carcinoma. And base on these indicators were used as positive control, the current study showed that in nasopharyngeal carcinoma patients that treated with cetuximab, those with KLF4 expression in the nucleus showed a poor prognosis, and the KLF4 expression in the nucleus was significantly associated with common drug resistance-related indicators such as H-Ras and PI3K mutations. One of the underlying mechanisms might be as follows; mutations in EGFR downstream pathways, including EGFR/cascades-Ras/Raf/MAPK and EGFR/PI3K/Akt/mTOR [25, 27], and among them, H-Ras mutation was dominant in cascades-Ras/Raf/MAPK pathway, while the PI3K mutation was dominant in PI3K/Akt/mTOR pathway. On the other hand, a mutual regulation between H-Ras and PI3K [28, 29], when KLF4 was affected by one of the mutated genes, might be affected by another gene mutation consecutively.

Presently, several studies showed that the expressions of Ras [10] and PI3K [30, 31] were related to KLF4, and our study showed that the mutations in H-Ras and PI3K were associated with KLF4 in nasopharyngeal carcinoma. The in vitro experiments also confirmed that the mutations in H-Ras and PI3K did not exert a significant effect on the resistance of cetuximab when KLF4 was overexpressed in the cytoplasm, thereby indicating that cetuximab-mediated resistance by H-Ras and PI3K mutations was induced by the high expression of KLF4 in the nucleus. Nonetheless, this study had a limitation. The mutations in *H*-*Ras* and *PI3K* genes did not result in the high endogenous expression of KLF4 in the nucleus when KLF4 was specifically overexpressed in the cytoplasm, which might be attributed to the competitive expression of proteins in subcellular localization [32], and the specific mechanism needs to be elucidated further.

The role of KLF4 in nasopharyngeal carcinoma attracted increasing attention. The current study found that the detection of KLF4 expression in the nucleus by IHC could predict whether the nasopharyngeal carcinoma was resistant to cetuximab based on the previous study. Since the IHC detection of KLF4 was a simple and feasible clinical method, it could be used to predict the efficiency of cetuximab quickly. Furthermore, this study showed that the altered subcellular localization of KLF4 could affect the resistance to cetuximab, suggesting a way to block the localized expression of KLF4 in the nucleus to improve the efficacy of cetuximab in the treatment of nasopharyngeal carcinoma as well as head and neck tumors.

## Conclusions

In conclusion, our results revealed that aberrant nuclear Klf4 expression can lead to cetuximab drug-resistance of nasopharyngeal carcinoma patients. The evaluation of the nuclear Klf4 can be a useful biomarker in determining the prognosis of NPC patients. Meanwhile, c-MET, nEGFR over-expression, H-Ras, and PI3K mutations could be as biomarker for cetuximab resistance in nasopharyngeal carcinoma.

## Abbreviations

Klf4: Kruppel-like factor 4
cKlf4: cytoplasm Kruppel-like factor 4
nKlf4: nuclear Kruppel-like factor 4
EGFR: epithelial growth factor receptor
nEGFR: nuclear epithelial growth factor receptor
PI3K: phosphoinositide 3-kinase
mPI3K: mutation phosphoinositide 3-kinase
H-Ras: harvey rat sarcoma
m-Hras: mutation Harvey rat sarcoma
c-Met: tyrosine-protein kinase Met
PTEN: phosphatase and tensin homolog
NES: nuclear export signal
NLS: nuclear localization signals
EB: Epstein–Barr
HPV: human papillomavirus
ZFs: zinc fingers
erbB-2: human epidermal growth factor receptor 2
IHC: immunohistochemistry
arms-PCR: arms quantitative real-time PCR
SDS-PAGE: sodium dodecyl sulfate–polyacrylamide gel electrophoresis
ECL: enhanced chemiluminescence
HR: hazard rate
CI: confidence interval
OS: overall survival
